# Impact of Ultra-High-Dose-Rate Irradiation on DNA: Single-Strand Breaks and Base Damage

**DOI:** 10.3390/ijms26051800

**Published:** 2025-02-20

**Authors:** Yucheng Wang, Yan Zhang, Chenyang Huang, Qibin Fu, Tuchen Huang

**Affiliations:** Sino-French Institute of Nuclear Engineering and Technology, Sun Yat-sen University, Zhuhai 519082, China; wangych89@mail2.sysu.edu.cn (Y.W.); zhangy883@mail2.sysu.edu.cn (Y.Z.); huangchy227@mail2.sysu.edu.cn (C.H.)

**Keywords:** ultra-high dose rate radiation, FLASH effect, single-strand break, base damage, plasmid concentration

## Abstract

Studying different types of DNA damage induced by ultra-high-dose-rate (UHDR) irradiation is essential for understanding the mechanism underlying the FLASH effect. pBR322 plasmid DNA was irradiated using an electron FLASH beam. The content of each subtype of plasmid DNA was measured via gel electrophoresis, and the extent of DNA double-strand breaks (DSBs) and single-strand breaks (SSBs) under UHDR and conventional-dose-rate irradiation (CONV) was quantitatively compared. Furthermore, by adding the endonucleases Nth and Fpg, the extent of base damage in the UHDR and CONV group was quantitatively analyzed. In addition, the effects of different plasmid concentrations on the damage degree were studied. The induction rates of SSBs (×10^−3^ SSB/Gy/molecule) under UHDR and CONV were 21.7 ± 0.4 and 25.8 ± 0.3, respectively. When treated with the Fpg and Nth enzymes, the base damage induction rates (×10^−3^ SSB/Gy/molecule) under UHDR and CONV irradiation were 43.3 ± 2.0 and 58.4 ± 4.5, respectively. Additionally, UHDR irradiation consistently reduced SSBs and base damage at both high and low plasmid concentrations, although the absolute level of DNA damage was still influenced by the plasmid concentration. UHDR has a significant effect on reducing SSBs and base damage when compared to CONV across plasmid concentrations.

## 1. Introduction

Preclinical studies have shown that ultra-high-dose-rate (UHDR) irradiation can significantly reduce radiation damage to normal tissues without compromising tumor control, a phenomenon known as the FLASH effect. The FLASH effect has been confirmed to occur for various types of ionizing radiation and different species and experimental systems [1,2,3,4,5,6]. However, the mechanism of the FLASH effect remains unclear. DNA damage, cytotoxicity, and the activation of cell death pathways form the basis of the biological mechanisms underlying the FLASH effect. Ionizing-radiation-induced nuclear DNA damage includes base damage, single-strand breaks (SSBs), double-strand breaks (DSBs), DNA cross-links, and clustered damage, which are the key causes of cell death and an important topic in radiobiology [7]. However, it is still unclear whether UHDR irradiation can reduce nuclear DNA damage. Therefore, studying different types of DNA damage after UHDR is of great significance for gaining a deeper understanding of the mechanism of the FLASH effect.

The impact of UHDR irradiation on nuclear DNA damage remains controversial. Shi et al. demonstrated via Western blot analysis that the expression levels of γ-H2AX and phosphorylated 53BP1 were nearly identical in intestinal crypt organoids exposed to FLASH and conventional-dose-rate irradiation (CONV) X-rays, suggesting that the effects of FLASH X-rays on genomic DNA damage were comparable to those of CONV radiation. They also confirmed that the 53BP1 foci induced by 4 Gy of UHDR irradiation in human intestinal epithelial HIEC-6 cells were similar to those induced by CONV irradiation using immunofluorescence detection [8]. However, Favaudon et al. observed different responses of 53BP1 to FLASH and CONV electrons in MRC5 and IMR90 human lung fibroblasts [9]. Despite the observation time (0–3 h) being consistent with that noted by Shi et al., there were differences in cell lines and radiation conditions. Favaudon et al. used electron beams with a pulse dose rate as high as 10^7^ Gy/s. Levy et al. also found that compared with a CONV electron beam, abdominal UHDR irradiation with a pulse dose rate of 4.0 × 10^5^ Gy/s could significantly reduce genomic DNA damage in small-intestinal-crypt stem cells when measured using γ-H2AX foci [10]. Using comet electrophoresis, Cooper et al. observed a reduction in UHDR-induced DNA damage in peripheral blood lymphocytes under hypoxic conditions [11]. Overall, these differences may be attributed to the use of different cell types, dose rates, pulse structures, and other physical conditions during UHDR irradiation.

While mitigating radiation-induced damage to normal tissues, FLASH-RT has also been shown to maintain tumor control in a manner comparable to CONV-RT in several tumor types, e.g., ovarian cancer [10], cutaneous lymphoma [1], and glioblastoma [12]. Consistent with the equivalent tumor cytotoxicity, similar DNA damage (γ-H2AX foci) was observed in the cancer cells [10].

Additionally, existing experiments and models indicate that a dose threshold is required to observe the FLASH effect [12,13,14,15,16]. Typical dose-escalation experiments reported that a FLASH-sparing effect could be observed for a single dose greater than 23.5 Gy for skin toxicity scores ranging from 1.5 to 3.5 [14]. Notably, the dose threshold depends on the selected end point because of the varying radiation sensitivity of different end points. At relatively large doses, commonly used markers for characterizing DNA damage at the cell level, such as foci and micronuclei formation, are prone to dose saturation effects. As a result, it is difficult to accurately measure the extent of DNA damage in irradiated cells. Moreover, it is also challenging to measure different types of DNA damage, such as base damage and SSBs. Therefore, choosing an appropriate biological system and measurement method is crucial when performing in-depth quantitative research on DNA damage under FLASH conditions.

In this study, we used plasmids as a classic simplified biological system for research, as such a system is commonly used in studies on the physico-chemical stage of radiation action as a simplified model of cellular DNA without involving cellular repair processes. Furthermore, plasmids have the advantages of being easy to manipulate and having a well-defined structure. Using an electron FLASH beam, we quantitatively studied the types and extent of DNA damage due to UHDR irradiation and explored the impact of the plasmid concentration on DNA damage, providing important evidence for clarifying the mechanism of DNA damage due to UHDR irradiation.

## 2. Results

### 2.1. Comparison of SSBs Between CONV-RT and FLASH-RT

**Figure 1:**
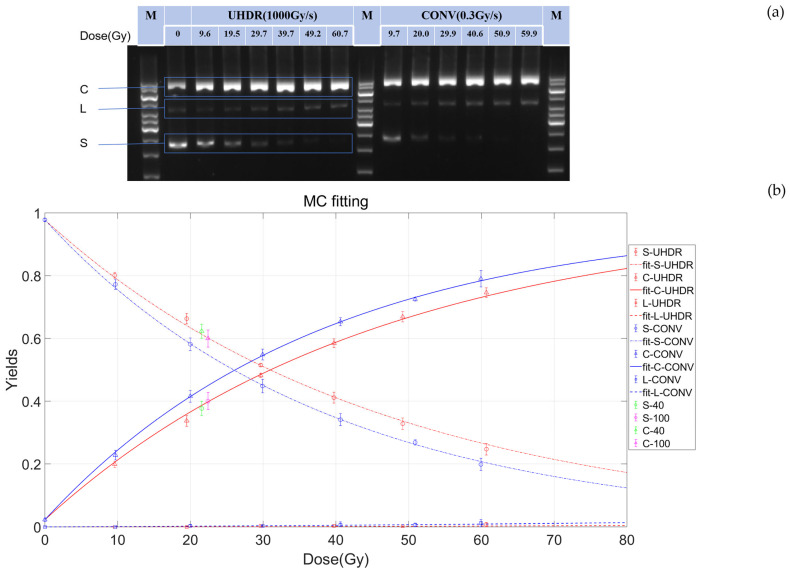
A quantitative analysis of the three structures of plasmid DNA after subjection to ultra-high-dose-rate (UHDR) and conventional-dose-rate irradiation (CONV) irradiation. (**a**) The gel electrophoresis of DNA damage at different irradiation doses. The left panel indicates the group treated with UHDR irradiation (1000 Gy/s), and the right panel indicates the group treated with CONV irradiation (0.3 Gy/s). The figure indicates open circular (C), linear (L), and supercoiled (S) DNA, as well as the molecular weight marker (M), separately. A full scan of the entire original image is shown in Supplementary Materials (Figure S1). (**b**) The fitting plot obtained using the McMahon and Currell method (Equations (4)–(6)) illustrated the trends of different yields varying with the irradiation dose under UHDR and CONV conditions. The dots represent the proportion of the three structures with respect to dose variation. The dash-dot, solid, and long dashed lines represent the predicted proportions of supercoiled, open circular, and linear structures for CONV (blue, 0.3 Gy/s) and UHDR (red, 1000 Gy/s), respectively. Green dots and pink dots represent the corresponding structures at dose rates of 40 Gy/s and 100 Gy/s, respectively. The error bars indicate the uncertainties in multiple measurements.

To systematically investigate the differences in DNA damage induced under FLASH and CONV conditions, we conducted gradient experiments with a dose range of 0–60 Gy using pBR322 plasmids as the subject. Standard plasmid DNA has three basic conformations: supercoiled (S), linear (L), and open circular (C). Since plasmids in different conformations have distinct types of electrophoretic mobility, bands can be observed at different positions after electrophoresis. Therefore, gel electrophoresis can be used to separate different subtypes of pBR322 plasmids and observe the changes in plasmid structure with respect to the irradiation dose. As shown in Figure 1a, after irradiation, the plasmids exhibited supercoiled (S), linear (L), and open circular (C) structures. Compared with the bands of non-irradiated plasmids, the intensity of the open circular structure band increased after irradiation, indicating a higher content of SSBs in the irradiated plasmids. As the radiation dose increased, the proportion of supercoiled DNA decreased, while that of open circular DNA increased, and the proportion of linear DNA remained relatively unchanged (Figure 1). The differences in the supercoiled structure band intensity between UHDR and CONV (Figure 1a) were analyzed using a two-tailed paired t-test, showing a significant reduction in plasmid breakage in UHDR conditions (*p* < 0.005).

To further quantify these observations, image analysis software and mathematical models were exploited to analyze the effects of UHDR and CONV irradiation on DNA subtypes quantitatively. First, we used ImageJ software to quantify the gel-imaging results and then employed Equations (4)–(6) to obtain the functions of the relative yields of DNA subtypes with respect to the radiation dose (Figure 1b). The yield of DSBs was very low, less than 2% of that of the SSBs (Figure 1b). In addition, the induction rate of SSBs induced by irradiation (expressed in units of ×10⁻³ SSB/Gy/molecule) for UHDR and CONV were 21.7 ± 0.4 and 25.8 ± 0.3, respectively, which are similar to the values obtained using Equations (7)–(10). The SSB induction rate for UHDR was 22.1, significantly lower than that for CONV (25.9) (*p* < 0.005) (Table 1), when calculated using Equations (7)–(10). This indicates that a UHDR can significantly reduce the induction of SSBs.

### 2.2. The Differences in Base Damage Under FLASH and CONV Conditions

Base damage is considered another major type of DNA damage induced by radiation. In the present study, the enzymes Fpg and Nth, which can recognize and excise damaged purine bases and pyrimidine bases, respectively, were utilized to convert DNA base damage into SSBs.

**Figure 2:**
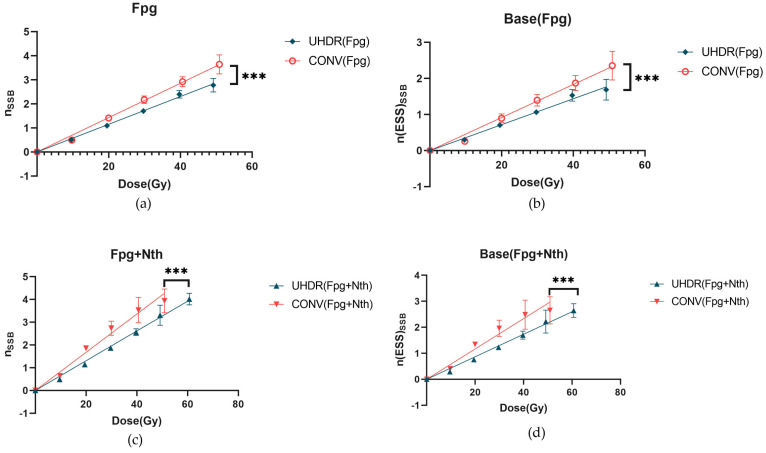
The effects of UHDR (1000Gy/s) and CONV (0.3 Gy/s) irradiation on DNA single-strand breaks (SSBs) and base damage. (**a**) The yield of SSBs per plasmid after treatment with Fpg enzyme as a function of irradiation dose. Red circles and blue diamond represent the CONV group and UHDR group, respectively. (**b**) The net yield of Fpg-enzyme-sensitive sites per plasmid after Fpg treatment as a function of the irradiation dose. (**c**) The yield of SSBs per plasmid after treatment with Fpg + Nth as a function of the irradiation dose. Red triangle and blue triangle represent the CONV group and UHDR group, respectively. (**d**) The net yield of Fpg + Nth-sensitive sites per plasmid after enzyme treatment as a function of the irradiation dose. Solid lines represent the fitted curves obtained via linear regression analysis of the experimental data. A *t*-test was used to compare the means of two related groups. *** indicates *p* < 0.0001, meaning that the differences are statistically significant.

The induction rates of SSBs induced by irradiation under different conditions were calculated using Equations (7)–(10). Following Fpg treatment, the induction rate of SSBs increased as expected. However, the SSB induction rate for UHDR remained lower than that for CONV (*p* < 0.005) (Figure 2a), indicating a protective effect. With an increasing irradiation dose, the difference in the yield of SSBs per plasmid ($n_{SSB}$) between the UHDR and CONV groups gradually increased. Further analysis using Equations (7)–(11) revealed that the induction rate of Fpg-sensitive sites via UHDR irradiation was significantly lower than that of CONV irradiation (*p* < 0.005), as shown in Figure 2b. As the dose increased, the difference in the net yield of enzyme-sensitive sites per plasmid (${n(ESS)}_{SSB}$) between the UHDR and CONV groups also became more pronounced. These results once again confirm that UHDR irradiation can significantly reduce not only SSBs but also base damage.

When the samples were treated with both Fpg and Nth, the rate of SSBs induced by UHDR irradiation was significantly lower than that of CONV irradiation (*p* < 0.005) (Figure 2c). Additionally, the calculated induction rate for base damage was also significantly lower than that for CONV irradiation (*p* < 0.005) (Figure 2d). The rates of DSBs induced by irradiation after enzyme treatment are listed in Table 1. The difference between the enzyme-treated and untreated samples indicates cluster damage. However, the proportion of the DSBs was too low, and it was associated with high-frequency errors. Therefore, further investigation was not conducted in this study. These data further indicate that UHDR irradiation had a significant effect in reducing SSBs.

### 2.3. The Effect of Plasmid Concentration on Radiation-Induced SSBs and Base Damage

**Figure 3:**
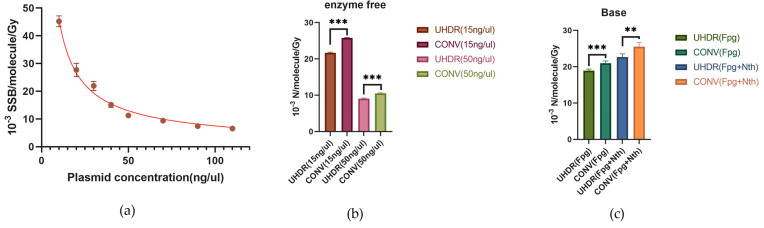
The effect of plasmid concentration on the yields of radiation-induced SSBs and base damage. (**a**) The rates of SSBs induced by irradiation in plasmid samples irradiated under CONV conditions (2 Gy/min) at different concentrations. The curves were fitted based on experimental data. (**b**) The rate of SSBs induced by irradiation in CONV (0.3 Gy/s) and UHDR groups (1000 Gy/s) at a concentration of 50 ng/µL or 15 ng/µL. (**c**) The induction rate of damage at Fpg (or Fpg + Nth)-sensitive sites induced by irradiation in CONV (0.3 Gy/s) and UHDR groups (1000 Gy/s) at a concentration of 50 ng/µL. A *t*-test was used to compare the means of two related groups. ***: *p* < 0.0001; **: *p* < 0.005.

As can be seen in Table 1, there were differences in the absolute values of SSBs among the different studies. This suggests that plasmid concentration may also play an important role in affecting the induction of SSBs and base damage via irradiation. Therefore, we investigated the influence of concentration on the induction rates of radiation-induced SSBs and base damage. As shown in Figure 3a, the induction rate of SSBs gradually decreased with an increasing plasmid concentration. In our previous experiments, we used a plasmid concentration of 15 ng/µL. For comparison, in this experiment, we used a plasmid concentration of 50 ng/µL. The results showed that compared with the 15 ng/µL concentration, the induction rate of radiation-induced SSBs was lower in the high-concentration samples (50 ng/µL) (Figure 3b). Consistently, the induction rate of SSBs in high-concentration plasmids in the UHDR group was still significantly lower than that in the CONV group, with a 14% reduction in SSB induction. Additionally, after treatment with Fpg or Fpg + Nth, the induction rate of damage at Fpg-sensitive sites in the UHDR group was 18.9 ± 0.9, significantly lower than that in the CONV group (21.0 ± 1.2) (*p* < 0.005). Moreover, the induction rate of damage at Fpg + Nth-sensitive sites in the UHDR group was also significantly lower than that in the CONV group (22.7 ± 1.7 versus 25.5 ± 2.2) (*p* < 0.005) (Figure 3c). The rates of SSBs induced by irradiation after enzyme treatment are listed in Table 1. These results suggest that while the plasmid concentration influenced the absolute level of SSBs and base damage, UHDR irradiation consistently reduced damage induction regardless of the concentration. This might have been because the total amount of free radicals produced by irradiation is limited. At higher plasmid concentrations, the close proximity of DNA molecules may reduce damage via competition for free radicals.

## 3. Discussion

UHDR irradiation is believed to significantly reduce radiation damage to normal tissues, but the mechanisms underlying the sparing effect remain unclear. Although several hypotheses, such as the DNA integrity hypothesis, the radical recombination hypothesis, and the immune hypothesis, have been proposed to explain the sparing effect [21,22], the impact of a UHDR on DNA damage is still a matter of debate [8,9,10,11,20,23]. In this study, we used plasmid DNA to investigate the effects of UHDR irradiation on different types of DNA damage. We found that compared to CONV irradiation, UHDR irradiation significantly reduced SSBs and preserved more polymeric structures. These findings are consistent with conclusions from other studies, although the types of beams used vary (protons [2,19], electron beams [20], and high-energy electron beams [17]). Although the induction rates of DSBs in the UHDR group were significantly lower than those in the CONV group (*p* < 0.005), the yield of DSBs was very low, and the variation error was large. Only a few studies have reported that UHDR irradiation can reduce DSBs [20].

To further investigate radiation-induced base damage, we employed enzymatic treatments. Two enzymes were primarily used in this study: Fpg and Nth. Both are bifunctional enzymes with N-glycosylase and AP lyase activities [24,25]. However, they differ in their specific functions: Fpg mainly recognizes and excises damaged purine bases, such as 8-oxoG and FapyG [24], while Nth targets damaged pyrimidine bases, including thymine glycol, 5,6-dihydroxythymine, 5-hydroxy-5-methylhydantoin, and uracil glycol [25]. Despite these differences, their distinct functions allow them to complement each other in the assessment of oxidative DNA damage. Fpg has only been used in one study [19], and no relevant FLASH studies have been conducted using Nth. Our results showed that compared to untreated samples, treatment with one enzyme increased the yield of SSBs because base damage could be converted into SSBs. Treatment with both enzymes produced more SSBs than treatment with Fpg alone due to the different cleavage sites of the two enzymes. Compared to CONV irradiation, UHDR irradiation produced fewer SSBs under the same dose conditions, regardless of whether one or both enzymes were used. Moreover, by subtracting the untreated group from the enzyme-treated groups, we observed that UHDR irradiation induced significantly less base damage under the same dose conditions. In living cells, base damage is mainly repaired through the base excision repair (BER) pathway. All oxidized base damage is removed from DNA by DNA glycosylases/AP lyase enzymes, which not only catalyze the removal of base damage but also cause strand breaks through β-elimination [26,27,28]. Fpg and Nth are two enzymes that play important roles in Escherichia coli [24,25], and their functional homologues (NEIL1, NEIL2, NEIL3 [29], and NTHL1 [30]) play important roles in human cells, making their application and an understanding of the DNA repair process in vivo relevant in this study.

In summary, compared to CONV irradiation, UHDR irradiation can reduce SSBs, base damage, and DSBs, with a significant reduction in SSBs and base damage. This may be because low-LET radiation (such as X-rays and electron beams) primarily induces DNA damage through free-radical-mediated indirect effects. Previous studies have proposed the radical recombination hypothesis and the radical recombination-antioxidant hypothesis. In our preprint work [31], for a specified dose, the fraction of radical recombination was determined to be dependent on the dose delivery time; that is, it was directly related to the dose rate. Collectively, unlike CONV irradiation, UHDR irradiation instantaneously generated high concentrations of peroxyl radicals, increasing the probability of peroxyl radical recombination and thereby reducing damage induced by peroxyl radicals [32]. Experiments have shown that different dose rates of irradiation produce similar quantities of H· and OH· radicals in aqueous solutions, but the content of lipid peroxides was reduced in the UHDR group [33]. The more compelling experimental evidence was that increasing the concentration of Tris solution reduced the induction rates of radiation-induced SSBs for both dose rates [17]. When eliminating radicals, the damage produced by the two dose rates was similar at the same dose [18]. Therefore, free radical recombination played an important role in reducing DNA damage under UHDR conditions.

Although our results support the radical recombination hypothesis, there is a need for more direct experimental evidence. A preprint work by our group sheds some light on this mechanism from in vivo experiments [31]. We found that the addition of the radical scavenger amifostine effectively eliminated the difference in mouse intestinal toxicity between FLASH and CONV groups [31]. However, the free radical levels, antioxidant levels, and their roles in DNA damage and repair still require testing in more experimental studies.

Furthermore, we compared the effects of plasmid damage among different published studies [2,17,19,20]. We found that there were certain differences in the absolute values of the radiation-induced SSB induction rates between different groups (Table 1). Although differences in radiation types and fitting methods contributed to these variations, our study revealed that the plasmid concentration during irradiation had a more significant impact. This phenomenon may be caused by the competition of high concentrations of plasmids for free radicals produced by limited irradiation. After increasing the plasmid concentration to 50 ng/µL, the radiation-induced SSB induction rate we obtained was lower and closer to that reported by Konishi et al. [19]. Despite the difference in the concentration ranges studied, the findings of Kong et al. also support this conclusion [34]. In the present study, to ensure accurate measurement of irradiation doses using EBT3 and EBT XD films, we reduced the plasmid concentration (15 ng/µL), thereby significantly lowering the dose required to achieve the same level of damage as would be induced by a high plasmid concentration (50 ng/µL). Additionally, recognizing the important role of free radical recombination in the FLASH sparing effect, we used PBS instead of Tris-EDTA buffer (TE buffer) to dilute the plasmids, reducing the Tris concentration in the plasmid solution to 0.3 mM. This lowered the radical scavenging rate of Tris, making the plasmid samples more susceptible to radical attack and increasing DNA damage at the same dose. Therefore, when comparing results from different research groups, factors such as plasmid concentration and Tris concentration in the plasmid solution should be considered.

All these results indicate that the pBR322 plasmid model is useful for understanding the effects of UHDR irradiation on DNA damage. However, it should be noted that studying the quantity and types of DNA damage are only the initial steps in understanding the biological effects of UHDR irradiation. Currently, the effect of UHDR irradiation on DNA repair, especially base excision repair pathways, is still unclear. This is also an important research direction with respect to understanding the cellular response to UHDR radiation. Furthermore, more research is needed on the subsequent impacts of UHDR irradiation, such as cell survival and tissue damage.

## 4. Materials and Methods

### 4.1. Sample Preparation

The pBR322 plasmid DNA solution (4361 bp, 0.5 μg/μL in 1 × TE) was purchased from Thermo Scientific (Vlinius, Lithuania; Beijing, China). More than 90% of it was in supercoiled circular form (form 1), and there was no linear form (form 3). Plasmid DNA solution was diluted in PBS buffer (1X, pH 7.4) to 15 ng/μL or 50ng/μL, and each DNA sample was prepared in 0.2 mL PCR tubes (Corning Cat. No. PCR-02-C) containing 50 μL of DNA solution for irradiation.

### 4.2. Irradiation

**Figure 4:**
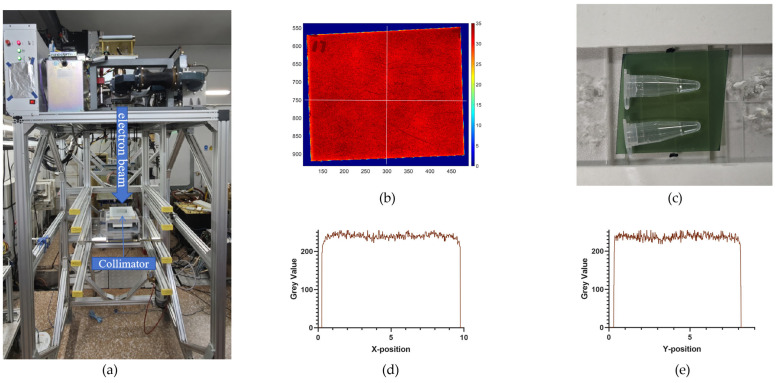
Schematic diagram of the electron beam irradiation facility and validation of its uniformity. (**a**) Schematic of the electron beam irradiation facility, where the electron beam was irradiated vertically downward from the top and formed an 8cm×8cm uniform irradiation field after passing through a collimator. (**b**) A film-scanning image showing the uniform irradiation field created by electron beam irradiation, demonstrating the consistency of the irradiation field. (**c**) A layout diagram of the samples, which were arranged in parallel, ensuring that the entire sample received a uniform radiation dose. (**d**,**e**) The grayscale value distribution of the film obtained along the horizontal and vertical white lines in Figure 4b, further confirming the uniformity of the irradiation field.

Irradiation experiments were performed with an electron linear accelerator, which provided a vertically irradiated electron beam with an energy of 6 MeV (Figure 4), at the Department of Engineering Physics, Tsinghua University, Beijing, China. The irradiation dose rate was altered by changing the Source-to-Surface Distance (SSD), pulse width, and pulse frequency. The formula for calculating the pulse dose rate is as follows: Pulse Dose Rate = Single Pulse Dose × Pulse Frequency. A pulse structure with a pulse dose rate of 10^6^ Gy/s, a single pulse of 3 Gy, and 333 pulses per second was applied in UHDR mode, while a pulse structure with a pulse dose rate of 10^5^ Gy/s, a single pulse of 0.05 Gy, and 6 pulses per second was applied in CONV mode. Prior to irradiation, the single pulse dose was confirmed using EBT 3 and EBT XD films. During irradiation, the film was placed beneath the sample, and the dose was calculated by reading the film 3 min after irradiation.

### 4.3. Enzyme Treatment

The Fpg working solution (from New England Biolabs, Ipswich, MA, USA; Beijing, China) and Fpg + Nth working solution were prepared using the enzymes Fpg and Nth, NEBuffers (for Fpg and Nth), recombinant albumin, and TE buffer (1X, pH 8.0) according to the protocol [24]. For samples requiring enzyme treatment (shown in Table 1), 10 µL of irradiated plasmid sample (pBR322 DNA: 150 ng) was withdrawn, and 1 unit of enzyme working solution was added. Subsequently, incubate the sample at 37 °C for 1 h, and then add 3 ul of EDTA solution (0.5M, pH 8.0) to terminate the reaction. Fpg recognizes oxidized purine bases, while Nth targets oxidized pyrimidine bases, making them complementary tools for assessing oxidative DNA damage [24,25].

### 4.4. Agarose Gel Electrophoresis and Quantification of DNA Strand Breaks

A total of 10 µL of plasmid sample (pBR322 DNA: 150 ng) was mixed with 5 µL of TE buffer (1X, pH 8.0) to dilute the sample to a concentration of 10 ng/µL. A total of 5 µL of the diluted plasmid sample (pBR322 DNA: 50 ng) was taken, and 1 µL of 6X TriTrack DNA loading buffer (Thermo Scientific, Beijing, China) was added, mixed well, and then subjected to electrophoresis. Electrophoresis was performed in a 1% agarose gel (pre-mixed with NA-Red) (Beyotime, Shanghai, China) in Electrophoresis Buffer (1X Tris Acetate-EDTA buffer) at 130V and 23 °C for 1 h. Under these electrophoresis conditions, SSBs that are less than six base pairs apart (one on each complementary strand) are detected as DSBs [35]. Subsequently, a fluorescent gel image was recorded, exported in the original TIFF format, and analyzed using ImageJ (v1.50i).

Image analysis involved manually identifying lanes using the rectangular selection tool to generate plots of pixel intensity versus position. The resulting curves contained peaks related to each band in the lanes. The area under each peak in the pixel intensity curve corresponded to the volume of the corresponding plasmid DNA isoform band. The total fluorescence intensity corresponding to the three forms of plasmid DNA was determined, namely, the three bands for supercoiled circular (no strand breaks, denoted as S), open circular (with SSB, denoted as C), and linear (with DSB, denoted as L) DNA. We normalized the fluorescence intensity of each band to the proportion of each DNA isoform by dividing the intensity of a single band by the sum of the intensities of the three bands in its lane according to Equations (1)–(3):

(1) $$S=\frac{S_{I}}{1.42S_{I}+C_{I}+L_{I}},$$

(2) $$C=\frac{C_{I}}{1.42S_{I}+C_{I}+L_{I}},$$

(3) $$L=\frac{L_{I}}{1.42S_{I}+C_{I}+L_{I}},$$

where $S_{I}$, $C_{I}$, and $L_{I}$ represent the peaks of forms 1–3 recorded in ImageJ (v1.50i), respectively.

### 4.5. DNA Damage Modeling

Using the robust curve-fitting model developed by McMahon and Currell [36], fractions of three forms were plotted as a function of the irradiation dose (Gy), where S, C, and L represent the fractions of supercoiled circular, open circular, and linear structures, respectively. $S_{0},\;C_{0},\;and\;L_{0}$ are the corresponding yields of supercoiled circular, open circular, and linear structures in the control sample, respectively. D is the dose, and $\beta_{S}$ and $\beta_{D}$ represent the rates of SSBs and DSBs induced via radiation (N/molecule/Gy):

(4) $$S=S_{0}exp(-(\beta_{S}+\beta_{D})D),$$

(5) $$C=exp(-\beta_{D}D)(C_{0}exp(-\frac{1}{2}{\beta_{S}}^{2}\rho D^{2})+S_{0}(exp(-\frac{1}{2}{\beta_{S}}^{2}\rho D^{2})-exp(-\beta_{S}D))),$$

(6) $$L=1-(C_{0}+S_{0})exp(-(\beta_{D}D+\frac{1}{2}{\beta_{S}}^{2}\rho D^{2})).$$

### 4.6. Base Damage Modeling

The yield of SSBs per plasmid ($n_{SSB}$) and DSB ($n_{DSB}$) and the net yield of enzyme-sensitive sites per plasmid (${n(ESS)}_{SSB}$) were calculated using the equations described by Povirk et al. [37]:

(7) S+C+L=1,

(8) $$n_{DSB}=L/(1-L),$$

(9) $$n_{SSB}=-ln(S)-ln(1+n_{DSB}),$$

(10) $$n_{SSB}=\beta_{S}D,\;n_{DSB}=\beta_{D}D,\;{n(ESS)}_{SSB}=∆\beta_{S}D,$$

(11) $${n(ESS)}_{SSB}={n(Fpg(or\;Nth))}_{SSB}-{n(enzyme\;free)}_{SSB},$$

where $\beta_{S}$, $\beta_{D}$, and $∆\beta_{S}$ represent the induction rates of SSB, DSB, and base damage caused by radiation (N/molecule/Gy), which were determined using linear regression in GraphPad Prism 8.0.2.

### 4.7. Statistics

In the present study, at least three independent replicate experiments were conducted for every experiment. The results are presented as the means ± standard deviations (SD). To determine significant differences between groups, we employed Student’s *t*-test and the F-test. Student’s *t*-test was used to compare the means of two related groups. The F-test was used to compare the variances between two groups of data to verify the assumption of the homogeneity of variances. Significant differences were determined using the *p*-value, with thresholds of *p* < 0.05 for significant differences and *p* < 0.005 for extremely significant differences.

## 5. Conclusions

Given the important role of DNA damage in cell survival and proliferation, radiation-induced DNA damage significantly affects tumor control and normal tissue complications. However, the effect of UHDR irradiation on DNA damage is still unclear. In the present study, through qualitative and quantitative analysis, it was found that UHDR irradiation significantly reduced SSBs in comparison to CONV irradiation. When treated with the enzymes Fpg and Nth, the induction rates of base damage in the UHDR group were also obviously lower than those in the CONV group. It should be noted that UHDR irradiation consistently reduced SSBs and base damage across the plasmid concentrations, although the absolute level of DNA damage was still influenced by the plasmid concentration. This study provides a better explanation for the different types of DNA damage induced by UHDR irradiation, which is of great significance for further understanding the mechanism of the FLASH effect.

## Figures and Tables

**Table 1:** Induction rates of single-strand breaks (SSBs) and double-strand breaks (DSBs) in different studies.

Radiation Quality	Energy (MeV)	Plasmid Concentration (ng/ul)	Dose (Gy)	Mean Dose Rate (Gy/s)	Enzyme	$\beta_{S}$ (×10^−3^ SSB/Gy/molecule)			$\beta_{D}$ (×10^−4^ DSB/Gy/molecule)		
						Mean	95%CI	*p*-Value (*t*-Test)	Mean	95%CI	*p*-Value (*t*-Test)
Electron *	6	15	0–60	UHDR	\	22.1	±0.4	<0.0001	0.9	±0.4	<0.0001
				CONV		25.9	±0.5		1.5	±0.6	
				UHDR	Fpg	57.7	±1.7	<0.0001	6.9	±1.3	<0.0001
				CONV		71.4	±2.5		10.2	±1.3	
				UHDR	Fpg+ Nth	65.4	±2.0	<0.0001	2.5	±1.5	<0.0001
				CONV		84.0	±4.6		12.8	±1.8	
		50	0–40	UHDR	\	9.0	±0.3	0.0004			
				CONV		10.5	±0.3				
				UHDR	Fpg	28.0	±1.2	<0.0001			
				CONV		31.5	±1.4				
				UHDR	Fpg+ Nth	31.7	±2.0	0.0003			
				CONV		36.0	±2.4				
Electron [17]	201	100	0–150	2E9	\	9.8	±0.4	\	2.6	±0.1	\
				0.08		13.3	±0.6		2.2	±0.3	
Electron [18]	9		0–30	125		10.9	±0.4		1.1	±0.4	
				0.05		11.8	±0.4		8.8	±0.4	
Proton [19]	59.5	50	0–65	48.6		12.5	±1.0		2.5	±0.2	
				0.057		16.9	±1.0		2.7	±0.4	
				48.6	Fpg	20.1	±1.2		4.3	±0.4	
				0.057		26.2	±1.4		5.0	±0.4	
Proton [2]	27.5	50	0–100	40	\	8.8	±0.3		1.1	±0.3	
				0.05		10.8	±1.3		1.2	±0.4	
Electron [20]	16	24	0–30	93.2		59.2	±4.9		5.4	±4.3	
				46.6		53.3	±3.1		5.4	±3.3	
				0.167		145.0	±27.4		8.1	±5.7	

Note: Our measured data were fitted to the plasmid DNA damage model described in Equations (7)–(10). The induction rates were obtained via linear fitting with respect to the dose, with the slope representing the induction rate. *: The present work.

## Data Availability

The data presented in this study are available within the article.

## Supplementary Materials

The following supporting information can be downloaded at https://www.mdpi.com/article/10.3390/ijms26051800/s1.
