# Supplementary material for: Impact of Ultra-High-Dose-Rate Irradiation on DNA: Single-Strand Breaks and Base Damage
Source: Int J Mol Sci. 2025 Feb 20;26(5):1800. doi: 10.3390/ijms26051800 (PMC11899290; doi:10.3390/ijms26051800)
Supplement: Supplementary file 1 [file ijms-26-01800-s001.zip › ijms-3473447-supplementary.pdf]

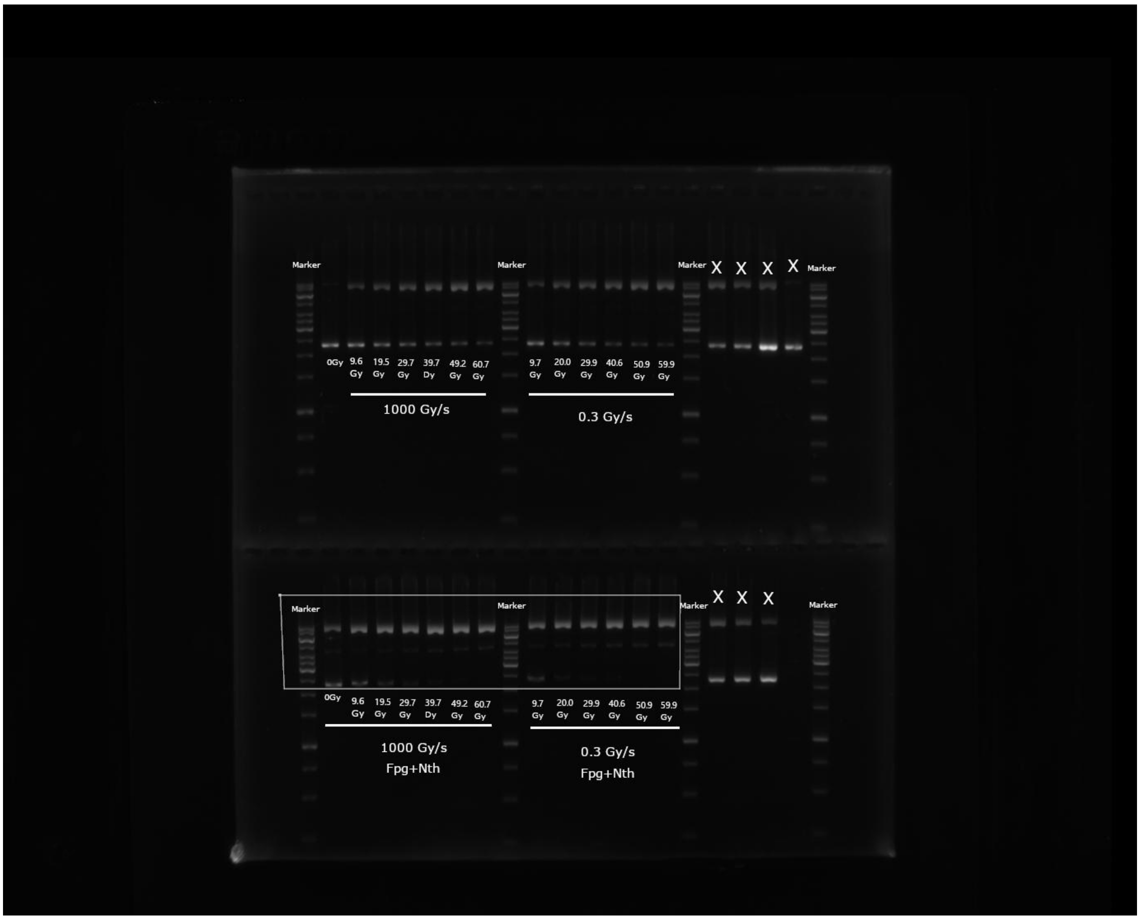

Figure S1. The full scan of the entire original image of Figure 1a, the parts displayed in Figure 1 have been boxed.
